# Marine reserves contribute half of the larval supply to a coral reef fishery

**DOI:** 10.1126/sciadv.adt0216

**Published:** 2025-02-05

**Authors:** Michael Bode, Severine Choukroun, Michael J. Emslie, Hugo B. Harrison, Jeffrey M. Leis, Luciano B. Mason, Maya Srinivasan, David H. Williamson, Geoffrey P. Jones

**Affiliations:** ^1^ School of Mathematical Sciences, Queensland University of Technology, Brisbane 4000, Australia.; ^2^ Securing Antarctica’s Environmental Future, Queensland University of Technology, Brisbane 4000, Australia.; ^3^ College of Science & Engineering, James Cook University, Townsville 4814, Australia.; ^4^ Centre for Tropical Water and Aquatic Ecosystem Research (TropWATER), James Cook University, Townsville 4811, Australia.; ^5^ Australian Institute of Marine Science, Townsville 4810, Australia.; ^6^ School of Biological Sciences, University of Bristol, Bristol BS8 1TQ, UK.; ^7^ Ecology & Biodiversity Centre, Institute for Marine and Antarctic Studies, University of Tasmania, Hobart 7001, Australia.; ^8^ Ichthylogy, Australian Museum Research Institute, Sydney 2010, Australia.; ^9^ Great Barrier Reef Marine Park Authority, Townsville 4810, Australia.

## Abstract

Marine reserves deliver impressive increases in the abundance and size of exploited species on protected reefs, but larval dispersal makes it difficult to estimate their wider benefits. Australia’s Great Barrier Reef (GBR) contains an extensive network of marine reserves. By combining GBR-wide fish surveys, larval dispersal models, and commercial fishery catch data, we calculate the system-wide ecological and economic contributions of these reserves for coral groupers (*Plectropomus* spp.), the region’s most important line fishery. Despite covering only 30% of reef habitat, the GBR’s marine reserve network contains half of the species’ biomass and generates most of its reproductive output (55%), half of the system’s larval settlement (50%), and almost half of the total fishery yield (47%).

## Teaser

Marine reserves generate more than half of coral groupers produced on the GBR and half of the fish caught by the fishery.

## INTRODUCTION

Marine reserve networks are primarily implemented to conserve species within (*1*, *2*) and sustain fisheries beyond (*3*, *4*) their boundaries. To achieve both these goals, a chain of events must occur. To start with, established marine reserves must support more abundant, larger fish with greater reproductive output than surrounding fished areas (*1*, *5*–*7*). Then, this increased supply of offspring must be spread among reserves and exported to the fished areas via larval dispersal (*4*, *8*, *9*). Last, for benefits to accrue to fisheries, enough larvae must be supplied to locations that are unprotected and targeted by the fishery (*3*, *10*, *11*).

Although the benefits of reserves to local protected populations are well established, the challenging task of proving the system-wide benefits to conservation and fishery yields has yet to be achieved. To do so, multiple layers of robust spatial data on abundance, size, reproductive output, and fishery catch must be available at a regional scale. A reliable biophysical model is also required to describe how larval dispersal will connect populations over hundreds of kilometers (*12*–*16*). Last, the data must be available over several years to account for the substantial temporal variation in each of these processes (*12*, *17*, *18*). In this study, we undertake each of these steps to estimate the ecological and economic contribution of a large network of no-take marine reserves to the larval settlement of coral grouper (*Plectropomus* spp., Serranidae), the most valuable line fishery on Australia’s Great Barrier Reef (GBR).

To estimate the local benefits of the GBR’s no-take marine reserves, we use a fishery-independent, multidecadal monitoring dataset on the biomass and size distribution of coral groupers, gathered across a biogeographically representative set of 133 reefs, both marine reserves and fished (*13*). We use a statistical model to extrapolate these observations to estimate the biomass on the remaining 2163 unsampled reefs. We integrate this abundance data with size-fecundity relationships (*14*) to estimate the total egg production of each reef.

Like most reef fishes, coral grouper metapopulations are demographically connected by dispersal during the larval stage rather than by adult movement (*15*, *16*, *19*). We therefore apply a high-resolution biophysical dispersal model for each new moon dispersal event across the analysis period. This model has been validated using a large genetic parentage dataset that was sampled from 18 reefs across 200 km of the southern GBR (*16*, *19*). The biophysical model predicts how the larvae produced on both fished reefs and marine reserves are exchanged across the metapopulation and, thus, the relative contribution of marine reserves to larval settlement on every reef. Last, we combine our predictions of larval settlement with spatial time series data on commercial harvests (*20*) to estimate the proportional contribution of marine reserves and fished areas to catches in the coral grouper fishery. See the Supplementary Materials for detailed information on these datasets.

These datasets capture demography, reproduction, larval connectivity, and commercial harvests, and we choose a series of years for which all are consistently available (2011 to 2013). The datasets extend across the entire GBR—more than 2300 km of coastline—which includes a network of 766 no-take marine reserves. This scale and resolution allow us to estimate the contribution of the marine reserve network to the entire coral grouper metapopulation and its fishery and also to estimate how this contribution varies through time and across space.

Ecological variables cannot be estimated across such a large spatial scale without uncertainty. We estimate the uncertainty associated with each dataset and use Monte Carlo sampling to integrate its joint effects into our final predictions. As well as our best estimates, we also report a range that encompasses (i) the temporal variation seen across years in each dataset and (ii) the model and parameter uncertainty surrounding the biophysical larval dispersal model, our estimates of coral grouper biomass, and the species’ allometry and phenology (Supplementary Materials).

## RESULTS

### Contributions of the marine reserve network

#### Biomass and reproductive output

**Fig. 1.**
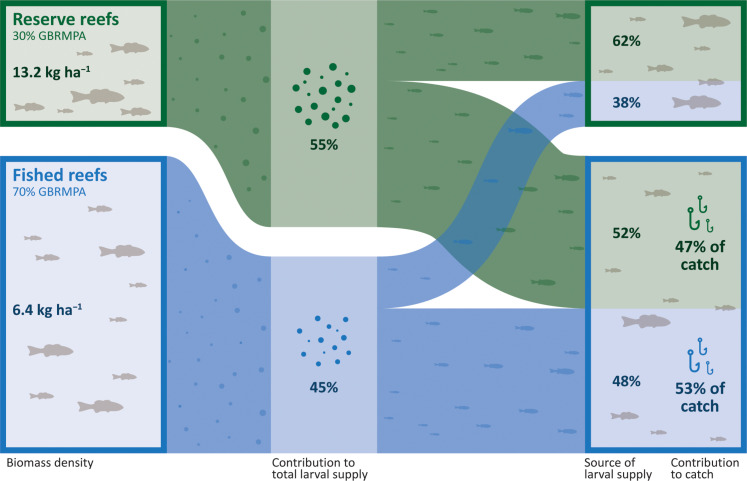
Relative contribution of the marine reserve reefs and fished reefs to the coral grouper metapopulation on the GBR. Despite protecting only 30% of coral grouper habitat, marine reserves support twice the biomass density of fished reefs (13.2 kg ha^−1^, compared with 6.4 kg ha^−1^) and produce 55% of total reproductive output. Marine reserves supply 52% of larvae that settle on fished reefs and 63% of larvae that settle on reserved reefs. Last, 47% of the annual commercial coral grouper catch by weight was spawned on marine reserves.

On the GBR, the marine reserve network protects less than a third of coral grouper habitat (30%; Fig. 1). However, these no-take marine reserves are home to 51% (47 to 54%) of the total coral grouper biomass (all ranges encompass 95% of both interannual variation and uncertainty). Marine reserves contain a disproportionate amount of the system-wide coral grouper biomass, because their biomass per unit area is 2.1 times higher (1.8 to 2.4 times higher) than on the fished reefs (fig. S2). This higher density is a result of their marine reserve status, which has increased coral grouper biomass on protected reefs, while trends on fished reefs over the same period have been generally stable (*13*).

Across all GBR populations, coral grouper in marine reserves are larger on average (45.4-cm versus 42.6-cm fork length), making them substantially more fecund (96,100 versus 69,800 batch fecundity). The combination of more numerous and larger fish in the marine reserves means that their total reproductive output per unit area is higher than that of fished reefs by a factor of 2.5 (2.0 to 3.1). Overall, accounting for the relative abundance and size of coral grouper on fished and protected reefs, we estimate that the 30% marine reserve network is responsible for 55% (50 to 60%) of total egg production across the GBR. By contrast, the 70% of reef habitat open to fishing is responsible for 45% (40 to 50%) of total egg production (Fig. 1).

#### Larval settlement and commercial catch

While the disproportionate reproductive output of the coral grouper population inside reserves is important, its contribution to the next generation of fish depends on larval dispersal patterns. Since larval dispersal is often dominated by the local retention of larvae (*21*, *22*), meaningful larval spillover from marine reserves to fished reefs cannot be taken for granted.

Our biophysical model of larval dispersal predicts that substantial spillover does occur and that marine reserves are responsible for 50% (45 to 54%) of all larval settlement across the GBR metapopulation: 62% (53 to 73%) of settlement into marine reserves and 52% (44 to 61%) of settlement into fished areas. These proportions mirror smaller-scale empirical studies in the southern GBR, which found that 28% of reef habitat in a network of marine reserves supplied 41% (±11% SD) of larval settlement (*17*). When the heterogeneous distribution of commercial catches is taken into account (fig. S7)—that is, the total catch from each reef varies between reefs and between years—we estimate that the marine reserve network is the source of 47% (37 to 54%) (by weight) of the coral grouper caught by the commercial fishery (Fig. 1).

## DISCUSSION

**Fig. 2.**
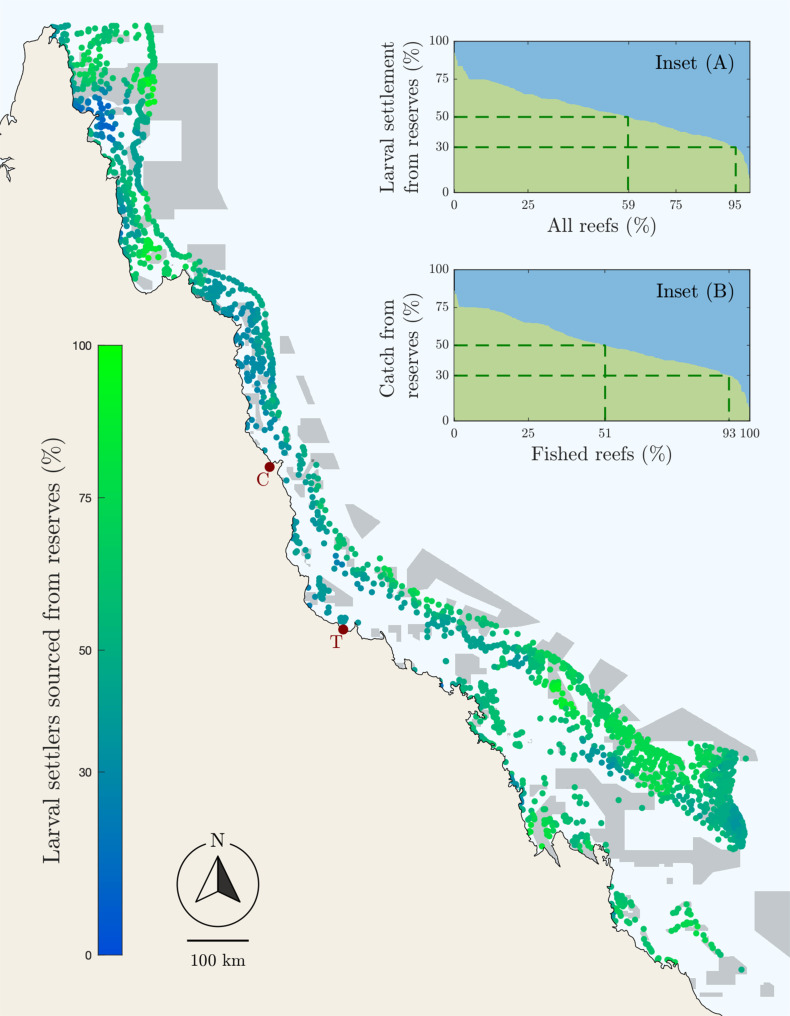
Spatial variation in the contribution of the GBR’s marine reserve network to larval settlement. Dots represent single reefs, colored by the mean proportion of larvae that were sourced from a marine reserve, between 2011 and 2013. The largest cities of Townsville (T) and Cairns (C) are shown. Inset plots show (**A**) the proportion of settlement and (**B**) the coral grouper commercial catch, which was generated by the marine reserve network, for each reef.

Marine reserves contributed to larval supply and catch throughout the GBR (Fig. 2 and fig S9). A total of 95% of reefs receive at least 30% of their larval settlement from reserves, and 59% of reefs receive more than half from reserves (Fig. 2A). A total of 93% of fished reefs receive more than 30% of their catch from reserves, and 50% of fished reefs receive half or more (Fig. 2B). The GBR’s marine reserve network was primarily designed to protect a representative cross section of biodiversity rather than manage its multiple fisheries (*23*). The disproportionate contributions of marine reserves to the coral grouper stock and commercial catches are therefore fortuitous. Nevertheless, deliberate choices by systematic conservation planners—to evenly distribute no-take marine reserves across the reef bioregions, for example (*24*)—are likely to be responsible for some of these system-wide achievements.

Our estimates of the egg production, larval settlement, and commercial catch produced by marine reserves are all uncertain. This uncertainty results from an incomplete understanding of the biological, ecological, and physical dynamics of the GBR but also from substantial year-to-year variation in coral grouper abundances, larval dispersal patterns, and the behavior of the commercial fishery. Nevertheless, even the lower end of our estimates confirms that the marine reserve network is a disproportionate contributor to the coral grouper metapopulation and fishery.

Some reefs receive almost all their larvae from reserves, while in some coastal areas, larval settlement from reserves is very low, particularly on midshelf reefs off the coast of the two largest cities on the GBR coastline: Cairns and Townsville (Fig. 2). This spatial variation can be attributed to a number of factors whose importance varies substantially in space. The size and spacing of reserves differ across the system (*25*), introducing variation in the size of source populations and the proportion of larvae dispersing to fished and reserve areas. Coral grouper densities are highest on southern reefs, and the biomass density difference between fished reefs and marine reserves is greatest on southern and inshore reefs (fig. S2), likely because these reefs attract the greatest concentrations of fishing effort (fig. S5). Last, these different drivers are then filtered through larval dispersal, which can connect reefs at large distances, in complex and asymmetric patterns. Because the strength of larval supply is a spatially variable combination of all of these factors, it is not useful to identify any single factor as most responsible for the disproportionate contribution of the GBR’s marine reserves.

Our results indicate that, while marine reserves reduce the area accessible to commercial fishers, they contribute one of every two fish to the fishery at present levels of exploitation. These results assume that the proportional composition of the settling larval cohort will eventually be reflected in the catch of legally sized adults. For example, if 50% of larvae that arrive on a particular reef come from marine reserves, then 3 years later, we assume that 50% of 3-year-old adults on that reef were spawned in reserves. This is based on the plausible and parsimonious assumption that post-settlement processes on fished reefs—particularly recruitment, growth, and survivorship—are independent of whether the larvae originated on marine reserves or fished reefs. It also makes the well-founded assumption that movements of adult *Plectropomus* spp. among reefs are rare (*14*, *20*).

Our results about the contribution of the marine reserve network to the commercial catch do not imply counterfactual predictions about fishing yields in the absence of those reserves. That is, they should not be taken as estimates of how much catches would change if the GBR were managed without its current network of marine reserves. To use our model to make such an estimate, we would need to make assumptions about the stock-recruitment relationship—that is, about how changes in larval supply rates translate to changes in adult abundance—as well as assumptions about how the commercial fishery would respond. We could not identify data that could defensibly parameterize these two relationships, which would be better approached using retrospective empirical analyses [e.g., (*13*, *26*, *27*)].

It is important to note that our results highlight the reproductive output of the fished reefs, as well as the marine reserves. If one of two of the fish caught by the commercial fishery is produced by a protected population, then the other one is produced by a fished population. Our results show that, on many reefs in the northern GBR (Fig. 2), considerably more than half of the fish caught are produced on fished reefs. Empirical studies on the GBR offer small-scale support for this result, finding that females on fished reefs are well represented in parentage datasets (*7*, *17*, *19*). The coral grouper fishery on the GBR is well managed using a combination of both marine reserves and traditional fishery management tools, including catch and effort restrictions, minimum size limits, and spawning closures. Stock levels are estimated to be 59% of unfished spawning biomass (*20*)—a sustainable level—and in some regions, the biomass density on fished reefs was comparable to marine reserves (e.g., Cooktown; fig. S2). This is a long way from the scorched-earth assumptions made by some models of marine reserves (*28*, *29*).

Networks of marine reserves have the potential to deliver a wide range of benefits to coral reef fisheries and ecosystems, but these require that a series of conditions be met. Our results show that, for the coral grouper fishery across the GBR, each of the links in this chain were functioning and connected. First, the exclusion of fishing mortality from marine reserves increases the density of adult fish. These larger and more abundant fish then go on to produce more eggs. Larval dispersal delivers these more numerous offspring to both fished and no-take zones across distances that range from a few hundred meters to hundreds of kilometers.

On the GBR, we estimate that these processes substantially amplify the contributions made by the marine reserve network. Together, they mean that 30% of protected reef habitat contributes 55% of the larval supply across the whole GBR, 50% of the overall larval settlement, and 47% of the commercial catch. Given that biomass on reserved reefs on the GBR is continuing to increase and populations on fished reefs are remaining stable (*20*), we expect these dual benefits to continue to accrue.

## METHODS

To estimate the benefits of the GBR’s no-take marine reserves, we connect datasets estimating coral grouper biomass and population structure, reproductive output, biophysical larval dispersal patterns, and commercial fishery catches.

Coral grouper populations were quantified on 133 reefs (5% of the 2296 reefs) by underwater visual census. For those reefs that were not surveyed, population size and structure are inferred using statistical regression. Reef-scale estimates of coral grouper biomass are transformed into maps of annual predicted reproductive output using allometric relationships. This reproductive output is then used to predict larval settlement on all reefs in the system using high-resolution biophysical dispersal models. Fishery outcomes are estimated by cross-referencing the sources of larval settlement with the distribution of commercial fishing effort, sourced from government logbook data. See the Supplementary Materials for full details.

## Supplementary Materials

### This PDF file includes:

Supplementary Materials and Methods Figs. S1 to S9 References
