# Supplementary material for: Marine reserves contribute half of the larval supply to a coral reef fishery
Source: Sci Adv. 2025 Feb 5;11(6):eadt0216. doi: 10.1126/sciadv.adt0216 (PMC11797529; doi:10.1126/sciadv.adt0216)
Supplement: Supplementary file 1 — Supplementary Materials and Methods Figs. S1 to S9 References [file sciadv.adt0216_sm.pdf]

Science Advances
AAAS

Supplementary Materials for

Marine reserves contribute half of the larval supply to a coral reef fishery

Michael Bode *et al.*

Corresponding author: Michael Bode, michael.bode@qut.edu.au

Sci. Adv. **11**, eadt0216 (2025)
DOI: 10.1126/sciadv.adt0216

This PDF file includes:

Supplementary Materials and Methods
Figs. S1 to S9
References

Supplementary Materials and Methods

Methods overview

We begin by describing the *Model domain* and the *Study species*. We then describe each of the datasets required to robustly estimating the contribution of an entire marine reserve network across multiple years:

- Marine reserves affect both the density and size structure of targeted fish populations. Both must therefore be measured inside and outside reserves. From 1995 to 2019, coral grouper (*Plectropomus spp.,* Serranidae) populations were quantified across the Great Barrier Reef (GBR) Marine Park by a program of systematic monitoring, which estimated coral grouper density and size structure using underwater visual census techniques. A total of 133 reefs (5% of the 2,296 reefs modelled in the system) were observed. These methods are described in the *"Coral grouper biomass on surveyed reefs"* section.

- Population size and structure must be estimated for those reefs that were not surveyed. We applied statistical regression techniques to predict biomass per unit area on all reefs across the system, based on a range of covariates that included each reef's reserve status, for the years 2011–2013. These methods are described in the *"Estimating coral grouper biomass on unsurveyed reefs"* section.

- Reef-scale estimates of coral grouper biomass must be transformed into maps of annual predicted reproductive output (i.e., total larval production) for the years 2011–2013, using allometric relationships, as described in *"Estimating reproductive output from each reef"*.

- This reproductive output is then transported to new reefs (or back to the natal reef) by larval dispersal. Phenological data is required to estimate the timing of dispersal, and biophysical models are required to estimate the exchange of larvae between each pair of reefs in the GBR Marine Park. We used a recently published biophysical larval dispersal model for coral groupers, which was validated by genetic parentage assignments. The model generates annual connectivity matrices for 2011–2013. We describe the larval dispersal model in *"Biophysical larval dispersal model"*.

- Proportional data on larval dispersal are integrated with estimates of reproductive output to calculate the proportion of larval supply to each reef that comes from reefs inside and outside marine protected areas. We describe this process in "*Estimating the source of larval supply*".

- Fishery impacts are estimated by cross-referencing larval supply estimates with the distribution of commercial fishing effort (2014–2018). This dataset lags the other datasets, since it takes between 3-5 years for a settling coral grouper to reach a legal size and enter the commercial fishery. We describe our analysis of this data, and our subsequent calculations, in "*Calculating the proportion of the catch spawned in marine reserves*".

- Each of these data sets contain uncertainty that affects our confidence in the predictions. We discuss our approach to measuring and reporting uncertainty in *"Accounting for uncertainty"*.

Model domain

We define the domain of the analyses to be the extent of the Great Barrier Reef (GBR) Marine Park, which extends from a northern boundary in the Torres Strait, at latitude 10° 40' 55" S, down to a southern boundary at 24° 29' 55" S, between the cities of Gladstone and Bundaberg (Figure S1). The hydrodynamic model domain is much larger in scale – encompassing a large part of the Coral Sea – to avoid the effects of boundary forcings on the GBR Marine Park section of the model (see *Biophysical larval dispersal model*).

The Great Barrier Reef is one of the Earth's largest coral reef ecosystems, spanning over 2,300 kilometres along the northeastern coast of Australia. It comprises more than 2,000 individual reefs and shoals and 600 islands that extend across the continental shelf. As a southerly offshoot of the Coral Triangle, extending almost to the subtropics, the diversity of species on the GBR is not as high as other parts of the Indo-Pacific, but it has relatively high levels of endemicity.

The Great Barrier Reef Marine Park (GBRMP) is a federal Marine Park, first gazetted in 1975 to protect the GBR from petroleum exploration. The GBRMP is a single large protected area, which is partitioned into smaller areas where different types of activities are allowed ("different zones"). The highest levels of protection are no-take marine reserves, designated as either Preservation, Marine National Park, or Scientific Research zones, where commercial and recreational extractive activities are prohibited. We group these zone types together as "marine reserves" throughout. The first phase of zoning on the GBR, implemented between 1975 and 1992, protected approximately 5% of reef habitat. In 2004, following an extensive systematic conservation planning process, the GBR was rezoned into its current state (*23*), with approximately 30% of reef habitat now placed in no-take marine reserves (Figure S1).

The number of reefs within the GBR Marine Park varies according to the definition of "reef". We followed the Great Barrier Reef Marine Park Authority's (GBRMPA) official dataset, which defines 2,296 reefs. Of these reefs, 766 are no-take marine reserves, and 1,530 are open to various types of extractive activity. In the overwhelming majority of cases, marine reserves in the GBR Marine Park (known colloquially as "green zones" following GBRMPA's zoning maps) contain entire reefs, rather than parts of reefs. Both commercial and recreational fishing occur outside marine reserves, but both remain subject to a suite of species-specific regulations, including size limits, gear restrictions, bag limits, and annual total allowable catches.

Study species

In this study we focused on four congeneric species of coral grouper from the family Serranidae: *Plectropomus leopardus, P. laevis, P. areolatus,* and *P. maculatus*. Coral grouper are conspicuous predatory fishes on Indo-Pacific coral reefs and are preferentially targeted by fishers throughout their geographic range. On the GBR the species have overlapping geographic distributions, with *P. maculatus*, and *P. areolatus* being most abundance on the inshore reefs, *P. leopardus* being most abundant on midshelf reefs, and *P. laevis* being most abundant on outer shelf and oceanic reefs respectively (*30*). Two of the species, *P. maculatus* and *P. leopardus* are very closely related, sharing similar life history attributes and with the ability to hybridize; *P. laevis* are a longer lived species which attain a larger maximum size (*30–34*).

Coral groupers are the primary target of both the commercial and recreational sectors of the GBR line fishery, with an average annual commercial harvest of 983 metric tonnes between 2013 and 2018. *P. leopardus* comprise the large majority of the catch (*30*). For the present study we pooled the species to a *Plectropomus* spp. group, which we refer to collectively as coral grouper.

Coral grouper biomass on surveyed reefs

Coral grouper biomass was estimated by surveys undertaken by the Australian Institute of Marine Science's (AIMS) Long-Term Monitoring Program (LTMP). Running for over 35 years, the LTMP is the most extensive and comprehensive record of coral reef ecosystem health anywhere in the world. As part of the survey, underwater visual census (UVC) surveys of reef fish species were conducted annually or biennially between 1995 and 2019 at 133 inshore, midshelf and outer shelf reefs throughout the GBR Marine Park. See references (*13*) for detailed descriptions of the UVC methods.

UVC surveys collect data on both the length and abundance of coral grouper *Plectropomus* spp. The 133 surveyed reefs were chosen across 15 sectors (*13*). Of these surveyed reefs, 69 were open to fishing and 68 reefs were no-take marine reserves (NTMRs). For inshore reefs, five replicate 50m by 6m belt-transects were surveyed parallel to the reef crest, each separated by at least 10m along 9-12m depth contours. The total length of each fish was estimated to 5 centimetre bins. For midshelf and outer shelf reefs, five permanently marked 50m x 5m belt transects were surveyed parallel to the reef crest, each separated by at least 10 m along the 6–9 m depth contour. The total length of each fish was estimated to the nearest centimetre.

We calculated the average biomass of coral grouper for each surveyed reef by averaging across transects, and standardising values to grammes of biomass per 250 m^2. The total length (L_T) of the fish was converted to fork length (L_F) using a known allometric relationship $L_F = \frac{L_T + 0.42}{0.86}$, (*32*). Fish mass was estimated from fork length using a von Bertalanffy weight-length relationship with coefficients $a = 0.008$ and $b = 3.2$ (*32,35*). Figure S2 shows the range of values for marine reserves and fished reefs in each section of the GBRMP. Biomass is observed to vary between 563 to 1280 g/250m^2 higher on marine reserves than fished reefs. Highest biomass is observed between 0 and 50km from the coast, South of 20°S in the Keppels at 3705 g/250m^2 in the marine reserve zones and 1649 g/250m^2 in the fished zones, an average followed by the inshore Whitsundays region with 2884 g/250m^2 in the marine reserve zones and 880 g/250m^2 in the fished zones.

Estimating coral grouper biomass on unsurveyed reefs

To calculate the contribution of the whole GBRMP marine reserve network, we needed to estimate the biomass of coral grouper on the unsurveyed reefs, as well as those surveyed by the LTMP. We used a boosted regression tree ensemble model. This is a predictive statistical model, composed of multiple regression trees. The model was trained using the surveyed averaged biomass per reef, reef sectors, or reef groups, with the g/250m^2 of coral grouper used as the response variable. Continuous and categorical predictor data were as follows: (1) Cross-shelf variability: cross-shelf position (inshore, off-shore and mid-shelf), shortest straight-line distance to the coast of each reef (in km). (2) Latitudinal variability. (3) Annual variability as the year surveyed. (4) Zoning as marine reserve or fished. While cover coral, cyclones and bleaching events may also have a strong influence on coral grouper biomass, we chose to only use predictors that have known values for each reef.

The model was run using in MATLAB R.2019b, using the *fitrensemble* function, with the automatic hyperparameter optimization set as *Bayesian optimisation*. It was run 100 times, with 500 learning cycles applied each time. At each learning cycle, the software trains one weak learner for every 5 learners, in our case tree, consequently training 500*5=2500 learners. The software composes the ensemble using all trained learners. The model was chosen as to have the best fit and the smallest RMSE.

The model produced estimates of biomass that are in reasonable agreement with hold-out validation data (Figure S3). For reefs with moderate coral grouper biomass (i.e., above the 25% quantile of 250 g 250 m^{-2}), the relative error falls between 0.5 and 1.5. Distance to Coast was the most important predictor, followed by normalised latitude, shelf position, period, and zoning. We note that these results are based on cross-validation analysis among a relative small number of reefs in a very large system. However, the reefs surveyed by the LTMP were chosen to be representative of the GBR's biogeographic variation.

Estimating reproductive output from each reef

Allometric relationships: We estimated total reproductive output from individual reefs following the methods described in the most recent coral grouper stock assessment (*30*). We began by estimating the length-frequency distribution for each reef in the system. For those reefs that were surveyed by the AIMS LTMP, this length-frequency data was directly available. The LTMP measures the total length (L_T) of the fish, which we convert to the fork length (L_F) as described above. We hereafter only refer to fork length, which we simply denote as L. For those reefs that were not surveyed, we estimated the length-frequency distribution using a weighted sum of all nearby reefs of similar marine reserve status.

Let the relative frequency of length class c on reef i in timestep t to be $L_i^x(t)$, with $\sum_x L_i^x(t) = 1$. Let $\mathbf{S}(t)$ be the set of sampled reefs in timestep t with the same zoning as reef i (e.g., marine reserve, or fished reef). If reef i is not in $\mathbf{S}(t)$, then we would estimate its length-frequency distribution values to be a spatially-weighted average of the nearest reefs from the same zone:

$$L_i^c(t) = \sum\nolimits_{j \in \mathbf{S}(t)} L_j^c(t)\, e^{-0.015 d_{ij}}$$

Equation S1

The exponential distance weighting factor $e^{-0.015 d_{ij}}$ is based on estimates of ecological spatial autocorrelation on the GBR, given in reference (*36*). Once the value of $L_i^c(t)$ is calculated for all x, we normalize the values by dividing through by their total.

Given the estimated biomass of coral grouper on a given reef (see section above), we can calculate an estimate of $N_i^c(t)$, the abundance of fish in length class c on reef i in timestep t. From these estimates, we estimate $T_i(t)$ the total egg production on each reef in the system to be:

$$T_i(t) = d_i(t) \sum_c 0.73 \left(0.008 \left(L_i^c(t)\right)^{3.2}\right)^{1.6} \cdot N_i^c \cdot r_c.$$

Equation S2

The composite allometric relationship in Equation S2 is based on calibrated relationships between coral grouper mass and batch fecundity, which have exponent 1.6 and coefficient 0.73 (*37*), and calibrated relationships between coral grouper length and mass, which have exponent 3.2 and coefficient 0.008 (*33*). The uncertainty around these parameter values is not reported, and so we evaluate the consequences of allowing them to vary by $\pm 5\%$ (Figure S4). This may not seem like a large relative error, but these uncertainties affect the parameter values of a highly nonlinear function, and therefore have a large effect. For

a 50 cm individual, for example, this level of uncertainty mean that egg production could vary by a factor of 10: between 5.5×10^4 and 5.6×10^5 eggs (note the logarithmic scaling on the y-axis in Figure S4).

The variables r_c denote the proportion of reproductively mature coral grouper in each length class, based on the Effects of Line Fishing study (*34*), reproduced in Figure S4. This report did not include confidence bounds around the relationship, and so we have added uncertainty equivalent to $\pm 25\%$ around the mid-point of the relationship (i.e., for a fork length of 30cm, we estimate that between 45% and 70% of adults are reproductively mature, with a best estimate of 55%).

These allometric relationships capture the effect that adult size has on the number of eggs produced by a mother. Larger mothers also tend to produce eggs that contain more energy (*38*). However, it's not clear how much (if at all) this additional energy contributes to the survival of offspring during the larval stage or during recruitment. If the effect on survival is positive, this would tend to make our conclusions about the contribution of marine reserves a underestimate.

Coral groupers are sequential hermaphrodites, with a frequency-dependent transition from female to male. We follow the Queensland Department of Agriculture and Fisheries' stock assessment model for coral groupers (*30*), which makes the standard assumption about egg production in sequential hermaphrodites: that since sex ratios are socially controlled, the sexes should be combined in the model, and the sex ratio as a function of age not included.

Model of spawning phenology: Finally, the variable $0 \leq d_i(t) \leq 1$ indicates the proportion of adults on reef i that spawn in timestep t. Coral grouper spawning occurs throughout the year, but varies in intensity across the months and across different latitudes. On southern reefs, spawning is concentrated in the late summer, between February and May; in more northern latitudes, it is concentrated in the late spring and early summer (Figure S5). This information is taken from otolith reconstructions of spawning times undertaken in the northern GBR by (*37*) and in the southern GBR by (*17*).

Since data on spawning times was not available across the length of the GBR, spawning timing in unsampled areas was taken to be a linear interpolation between the central distribution (at latitude 17 south) and the southern distribution (at latitude 23 south). Spawning timing north of the central section was taken to be equivalent to that in the central section. To include this uncertainty in our estimates, we repeated our analyses multiple times, with the location of the central point varying at random between Lizard Island (latitude 14.5 south) and reefs off the Whitsundays (latitude 19.5 south).

Biophysical larval dispersal model

Three biophysical models were created for the locations and timing of the empirical parentage study, and biologically parameterized for the study species of coral grouper. Full details of the model are given in (*18*).

The three models shared a single hydrodynamic simulation model. The hydrodynamic model was based on a temporally-implicit 3D barotropic scheme, built from three nested computational grids with resolutions of 1.85 km (1 nautical mile) for the whole GBR, 370 m for the three sample regions, and 74 m around key reefs. The numerical scheme was developed from the models in (*39*) and (*40*). It incorporates a sub-grid scale parameterisation of hydrodynamic impedance around GBR reefs that results in more accurate modelling of currents passing through the complex matrix of reefs in the GBR lagoon. Currents were determined hourly throughout the period July 2011 to July 2013, which includes all dispersal events in the parentage dataset.

The biology of the larvae were simulated by an individual-based particle simulation model. For each release on the new moon, it tracked 25 million particles in 5 minute timesteps from their spawning as eggs to their settlement as competent larvae (or their mortality). Its behavioural assumptions incorporate the recommendations of (*41*), and include the buoyancy of pelagic eggs, realistic larval sensory ability, behaviour (ontogenetic vertical migration, swimming performance and orientation), pelagic larval duration and mortality, and adult spawning phenology. Importantly, all of these behaviours exhibit diel, spatial, and ontogenetic variation. They also exhibit individual variation, described using probability distributions, with the behaviour of each individual larva being sampled from these distributions.

Parameters were based on empirical data for larvae of *P. leopardus, P. maculatus* or the most closely-related grouper species for which the required information is known. For each new moon between July 2011 and July 2013, we simulated the release of 2.5×10^7 eggs across all reef slope habitats within the study domain, creating 24 biophysical dispersal matrices for each of the models (Figure S6). The elements $c_{ij}(t)$ of these matrices denote the proportion of the larvae produced on reef i that survived dispersal and settled on reef j, in spawning event t.

Three different variants of the biophysical larval dispersal model were created, and tested against genetic parentage data to validate their effectiveness in predicting larval dispersal patterns. These models differ in their descriptions of larval behaviour.

1. **Consistent Model**: This variant assumes a consistent behavioural pattern for each larva, throughout its development. Each larva's behaviour is modelled using a fixed set of parameters throughout its pelagic stage, with behaviours such as swimming depth and direction remaining constant. An individual's parameter values are sampled from a probability distribution. This model is based on the premise that there is natural variation in behaviour among individuals, but that this variation is maintained by an individual across time.

2. **Varying Model**: Unlike the consistent model, the varying behaviour model allows for more dynamic changes in larval behaviour at different developmental stages. This model exhibits the same distribution of behaviours across individuals seen in the Consistent Model, but in this case the individuals resample their specific parameter values a probability distribution for each behaviour trait at each timestep.

3. **Passive Model**: This model treats larvae primarily as passive particles, with their destinations mostly determined by ocean currents. In this model, larvae do not exhibit any active swimming behaviour or behavioural adaptations in response to environmental cues, although they do settle on nearby reefs once competent, implying some active and directed locomotion near the end of their PLD. For reef fish, whose swimming abilities are substantial and well-established, the passive model is often used as a baseline to evaluate the impact of including more complex larval behaviours, or to understand the role of physical oceanographic processes alone in shaping dispersal patterns.

The three models were validated for coral grouper using a contemporaneously sampled genetic parentage dataset. The parentage dataset was collected from three distinct reef regions within the southern Great Barrier Reef: the Keppel Islands, the Percy Islands, and the Capricorn Bunker group. Adults and juveniles were sampled from 18 different reefs, separated by as much as 250 km of ocean. The final genetic parentage data consisted of 69 identified parent-offspring relationships from a sample of 880 adults and 1,190 juveniles. It provided a direct measure of larval dispersal patterns, which were used to validate the predictions made by the biophysical models. The validation process used likelihood methods to evaluate the models, as well as assessing the model's capability to predict both the scale and direction of larval dispersal.

The comparison demonstrated that the three models could replicate observed dispersal events. The Consistent Model demonstrated the best performance, providing a statistically good match to the genetic parentage data. It reproduced larval patterns at both local and regional scales, and provided particularly accurate estimates of self-recruitment. The Passive Model, which treated larvae as neutrally buoyant particles influenced only by ocean currents, did not perform as well, but did offer a reasonable reproduction of observed dispersal patterns. Finally, the Varying Model provided the worst fit, failing to capture some critical aspects of larval dispersal as effectively as the consistent model.

Direct empirical validation of larval dispersal models is challenging because larval dispersal events are so difficult to observe, and data collection and analysis requires years of multidisciplinary effort. These results represent the most empirically substantive, largest-scale, and statistically rigorous effort to validate of larval dispersal models undertaken to date. However, it remains constrained by limited empirical data and regional scope, focusing solely on the southern GBR. In this model, we apply the models across the entire GBR to predict the network-wide contributions of marine reserves. In doing so, it is important to acknowledge that our predictions for the northern regions of the GBR will carry more uncertainty than those for the south.

Estimating the source of larval supply

We aimed to estimate the proportion of coral grouper larval supply to reefs across the GBR Marine Park that were spawned within the marine reserve network. The total amount of eggs produced on reef i at each spawning event t, $T_i(t)$, is dispersed throughout the GBR according to the biophysical connectivity matrices from the appropriate time period (i.e., for the corresponding monthly new moon). Let **A** be the set of all reefs in the system, let **R** be the set of reefs that are in marine reserves, and let **F** be the set of reefs that are open for fishing.

Total egg production across the whole GBR is estimated to be:

$$T_T = \sum_{i \in \mathbf{A}} T_i$$

Equation S4

The proportion that was generated by marine reserves is:

$$T_R = \frac{1}{T_T} \sum_{i \in \mathbf{R}} T_i$$

Equation S5

While the proportion that was generated on fished reefs is:

$$T_F = \frac{1}{T_T} \sum_{i \in \mathbf{F}} T_i$$

Equation S6

The total amount of larval supply to all reefs in the system is:

$$\Gamma(t) = \sum_{i \in \mathbf{A}} \sum_{j \in \mathbf{A}} T_i(t) \cdot c_{ij}(t)$$

Equation S7

The total amount of larval supply to all marine reserves in the system is:

$$\Gamma_R(t) = \sum_{i \in \mathbf{A}} \sum_{j \in \mathbf{R}} T_i(t) \cdot c_{ij}(t)$$

Equation S8

The total amount of larval supply to all fished reefs in the system is:

$$\Gamma_F(t) = \sum_{i \in \mathbf{A}} \sum_{j \in \mathbf{F}} T_i(t) \cdot c_{ij}(t)$$

Equation S9

The proportion of the larvae supply to all reefs that was spawned in marine reserves is:

$$\gamma_R(t) = \frac{1}{\Gamma(t)} \sum_{i \in \mathbf{R}} \sum_{j \in \mathbf{A}} T_i(t) \cdot c_{ij}(t)$$

Equation S10

The proportion of the larvae supply to a particular reef that was spawned in marine reserves is:

$$\gamma_{\mathrm{R}}^{j}(t) = \frac{\sum_{i \in \mathbf{R}} T_i(t) \cdot c_{ij}(t)}{\sum_{i \in \mathbf{A}} T_i(t) \cdot c_{ij}(t)}$$

Equation S11

The proportion of the larvae supply to all marine reserves that was spawned in marine reserves is:

$$\gamma_{RR}(t) = \frac{1}{\Gamma_{\mathrm{R}}(t)} \sum_{i \in \mathbf{R}} \sum_{j \in \mathbf{R}} T_i(t) \cdot c_{ij}(t)$$

Equation S12

The proportion of the larvae supply to all fished reefs that was spawned in marine reserves is:

$$\gamma_{RF}(t) = \frac{1}{\Gamma_{\mathrm{F}}(t)} \sum_{i \in \mathbf{R}} \sum_{j \in \mathbf{F}} T_i(t) \cdot c_{ij}(t)$$

Equation S13

Calculating the proportion of the catch spawned in marine reserves

To calculate the proportion of the total GBR catch that was spawned in marine reserves, we start by estimating $h_i(t)$, the total tonnes of coral grouper harvested from each reef i in year t. Coral grouper are targeted by recreational, commercial, and charter fishing vessels. Commercial vessels account for the vast majority of catch (829 tonnes in 2017-2018), compared to the recreational harvest (171 tonnes in 2017-2018) and charter harvest (82 tonnes in 2010, the most similar estimate) (*30*).

The Queensland Department of Agriculture and Fisheries report the total catch from the commercial coral grouper fishery in 30 nm x 30 nm grids across Queensland (Figure S7). These measurements are based on commercial logbook reporting, which is consistently available from 1988–2020. This data is licensed under CC BY-ND 3.0 AU, which allows new work to build upon the data without restrictions if attribution is made.

The spatial resolution of the catch reporting grids is larger than the GBR's individual reefs, and so we distributed the total catch in each reporting grid among the non-marine reserve reefs within that grid, proportional to their coral habitat area. Reefs that intersect more than one grid were attributed partial harvests accordingly. Those few reefs that are only partially a marine reserve are attributed harvests according to the area in their unprotected fraction.

For each reef i, we assumed that if a proportion x of the settling larvae came from marine reserves, then in subsequent years, the same proportion x of the catch was spawned in a marine reserve. Because it takes a number of years for juveniles to reach a length that is both legal to catch and commercial valuable, we need to offset the dates of the catch data that we use. The commercial fishery primarily targets small, legal individuals between 38 cm and 45 cm *(42).* These individuals fetch the highest prices per kilogram, and are more numerous. These individuals were likely spawned 3–4 years earlier than the date they were caught (Figure S8), based on their von Bertalanffy growth function (*35*).

We estimated that the proportion of the total catch across the GBR that was spawned in a marine reserve is:

$$F = \sum_{i \in \mathbf{F}} \mu_i \gamma_{\mathrm{R}}^i,$$

Equation S14

where μ_i is the proportion of the total catch for a given year that is caught on reef i.

Accounting for uncertainty

Our main results in the main text come from our best estimates of each of the parameters and datasets, and report an average across the three years for which comprehensive data is available. For each estimate, we also report ranges that include both the temporal variation observed in our datasets, and uncertainty around our various parameters and models.

To generate these ranges, we run 1,000 Monte Carlo replicates of the marine reserve contribution estimation process, with the inputs for each component of the analysis chosen at random from distributions that account for estimation uncertainty, and for temporal variation where possible. Specifically:

1. For each replicate, we choose a different year from the biomass dataset (i.e., temporal variation), and then vary the estimates on each reef by the amount of variation shown in Figure S3 (i.e., uncertainty).

2. For each replicate, we allow the allometric relationship between size and egg production to vary within the range shown in Figure S4 (uncertainty), and allow the relationship between size and reproductive maturity to vary within the range shown in Figure S4 (uncertainty).

3. For each replicate, we vary the relationship between spawning timing and latitude according to the range of possible values discussed in Figure S5 (uncertainty).

4. For each replicate, we vary the distribution of commercial catch among the observed years, as shown in Figure S7.

5. For each replicate, we choose at random from one of three biophysical larval dispersal models, which differ in their assumptions about the behaviour of coral grouper larvae. To generate our main estimates of reserve contribution, we used the specific biophysical larval dispersal model – called the "consistent" model – that had been validated with genetic parentage data from populations of *P. leopardus* and *P. maculatus* from the southern Great Barrier Reef *(18)*. Two other biophysical models were considered in those analyses, called "passive", and "varying". For each replicate in the sensitivity analyses, we chose one of these three models at random.

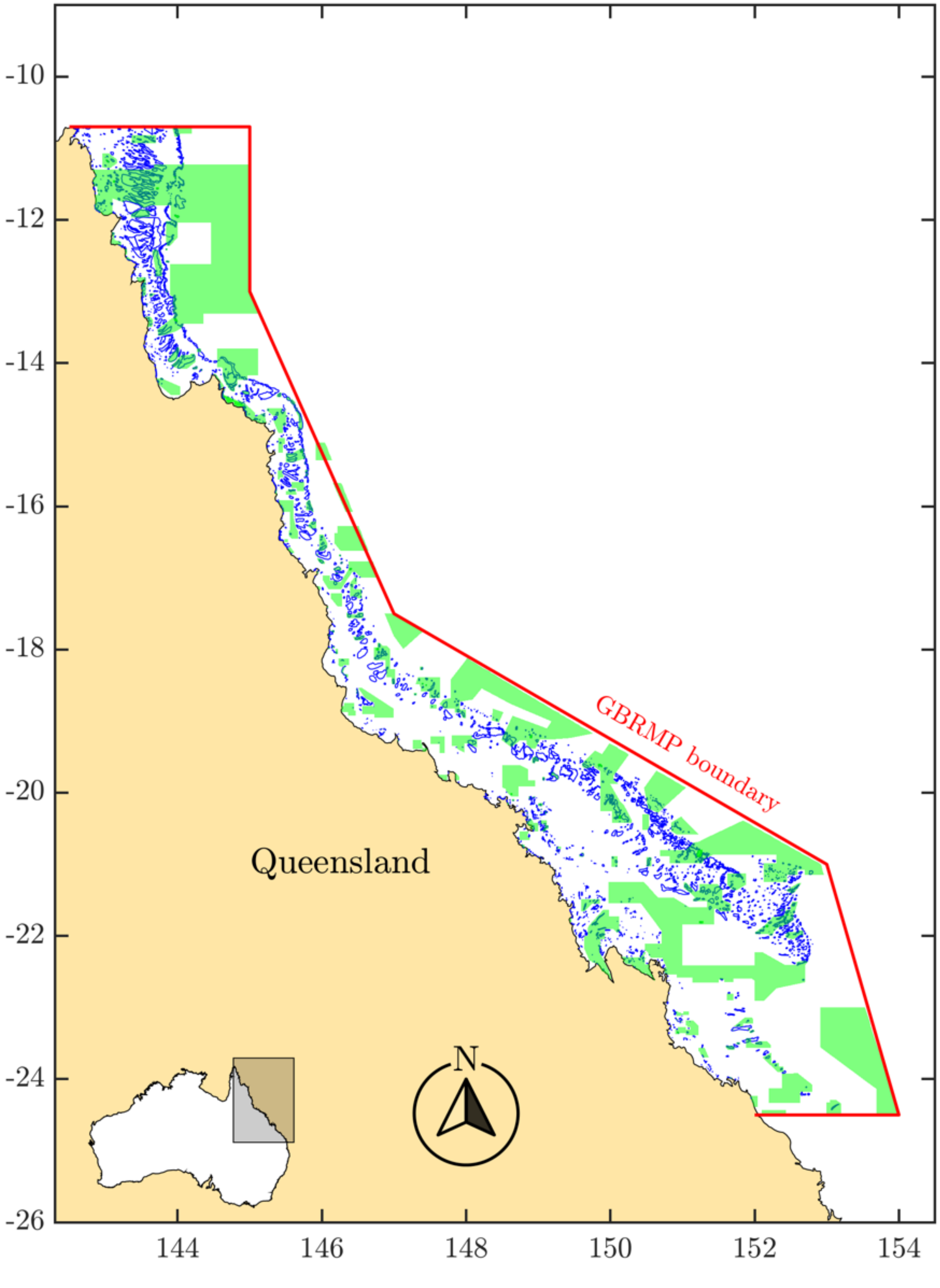

Figure S1: Map of Australia's Great Barrier Reef, showing the outline of the GBR Marine Park (red), the location of the coral reefs (blue), and the location of all no-take marine reserves (green shading). In this map, all no-take zones (Preservation, Marine National Park, and Scientific Research) have been coloured in green.

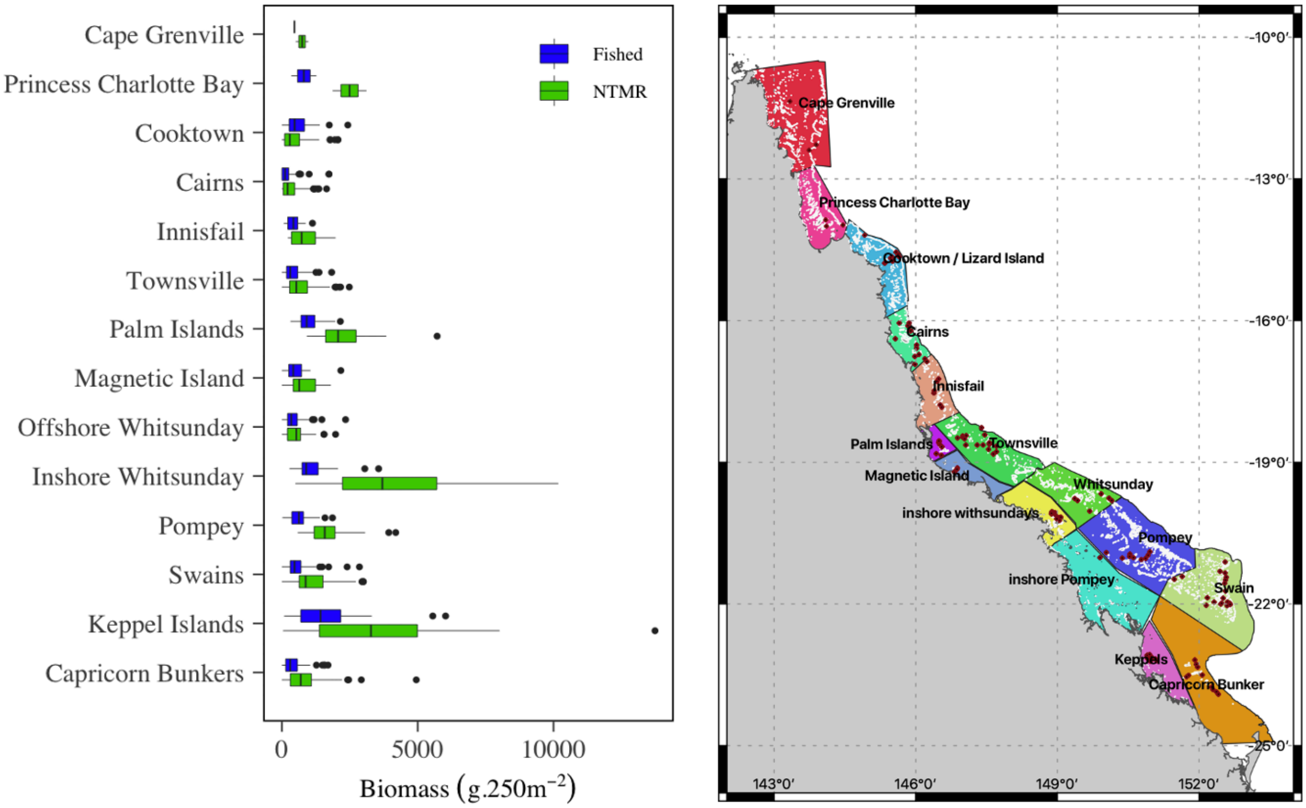

Figure S2: Left panel shows the range of *Plectropomus* spp. biomass across no-take marine reserves (green) and fished reefs (blue) in each sector of the GBR Marine Park. Averages are calculated from visual surveys from 1995 to 2019. Right panel shows the sector locations, with surveyed reefs in darker red. Values for the Inshore Pompey sector is combined with the Pompey sector. No-take marine reserves are labelled NTMRs.

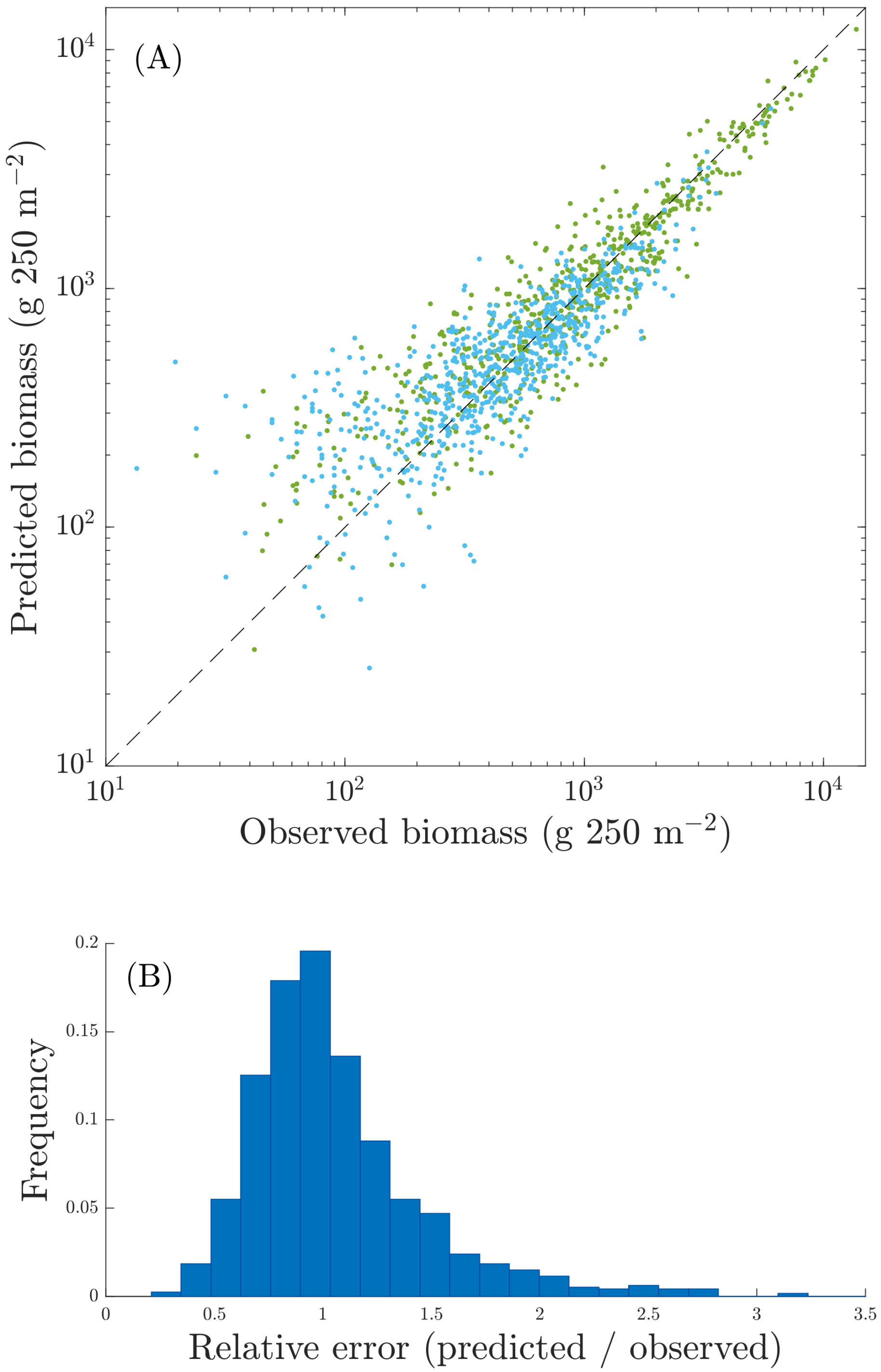

Figure S3: (A) Comparison between predicted and observed coral grouper biomass per 250m2. Each dot represents a reef monitored by LTMP, Inshore Monitoring Program between 1995-2019. Light blue dots indicate fished reefs; green dots indicate NTMR reefs. X-axis indicates the observed biomass on that reef in that year, and y-axis indicates the biomass predicted by the boosted regression tree model in a single-point hold-out validation. (B) frequency distribution of relative error in these predictions, for reefs with moderate coral grouper biomass (i.e., above the 25 percentile).

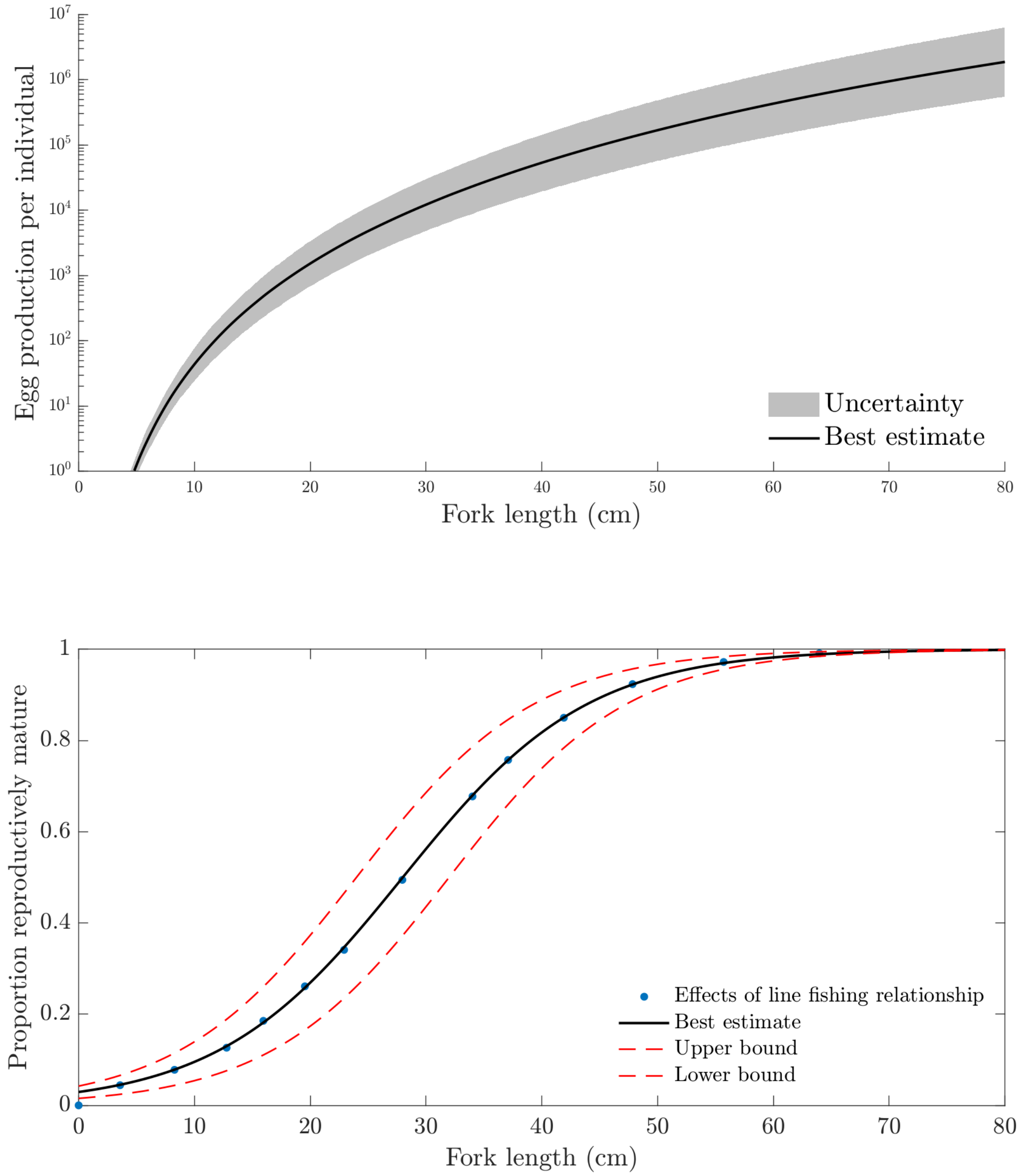

Figure S4: Allometric relationships incorporated into the estimates. Upper panel shows the number of eggs produced per year by females with a given length. Lower panel shows the relationship between coral grouper fork length (x-axis) and the proportion

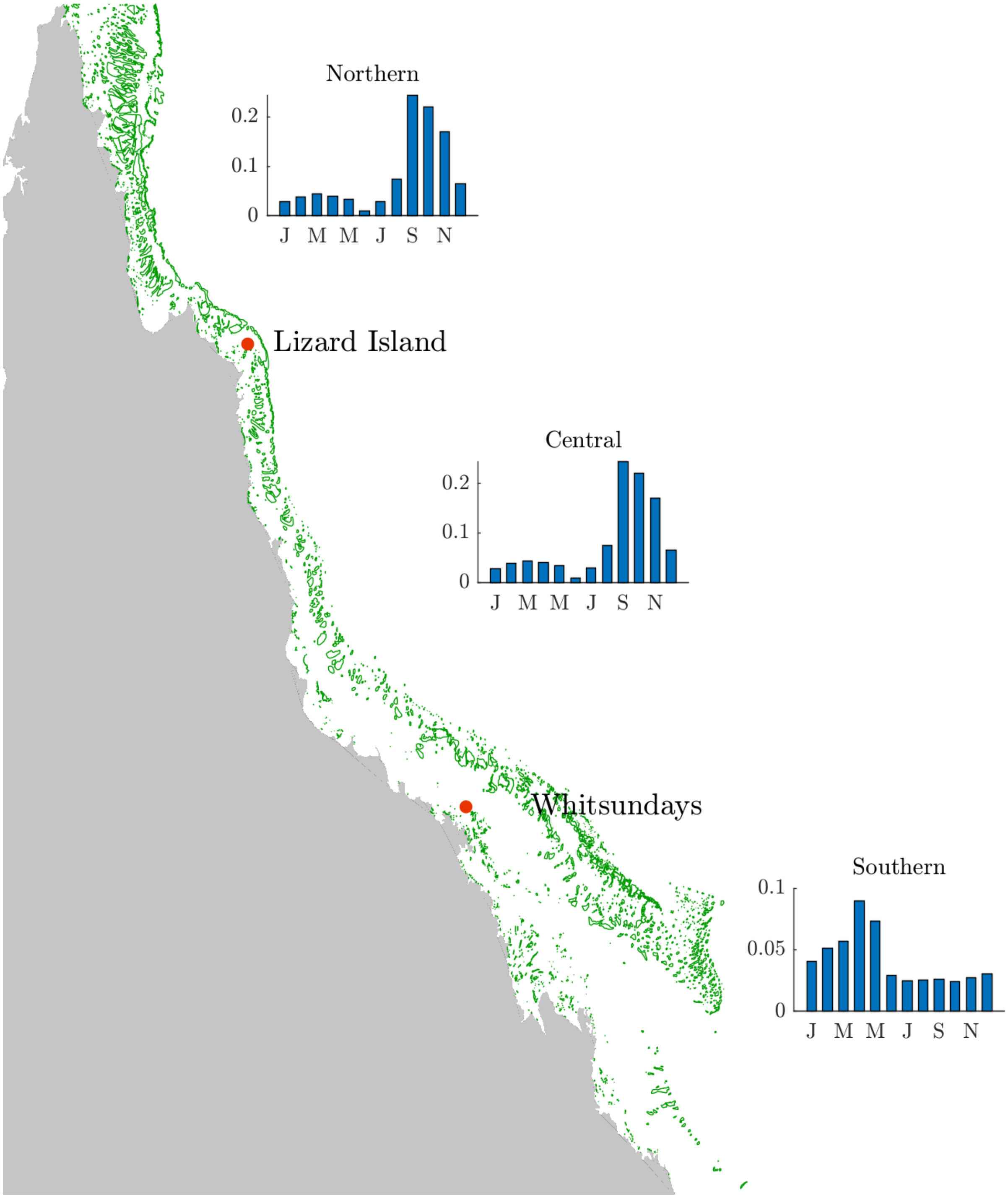

Figure S5: Spawning probability density across the months of the year (normalized) based on studies undertaken in the central and southern GBR. Northern values replicate the central data. The latitude at which the switch between northern/central spawning behaviour and southern spawning behaviour occurs is allowed to vary between the Whitsundays and Lizard Island.

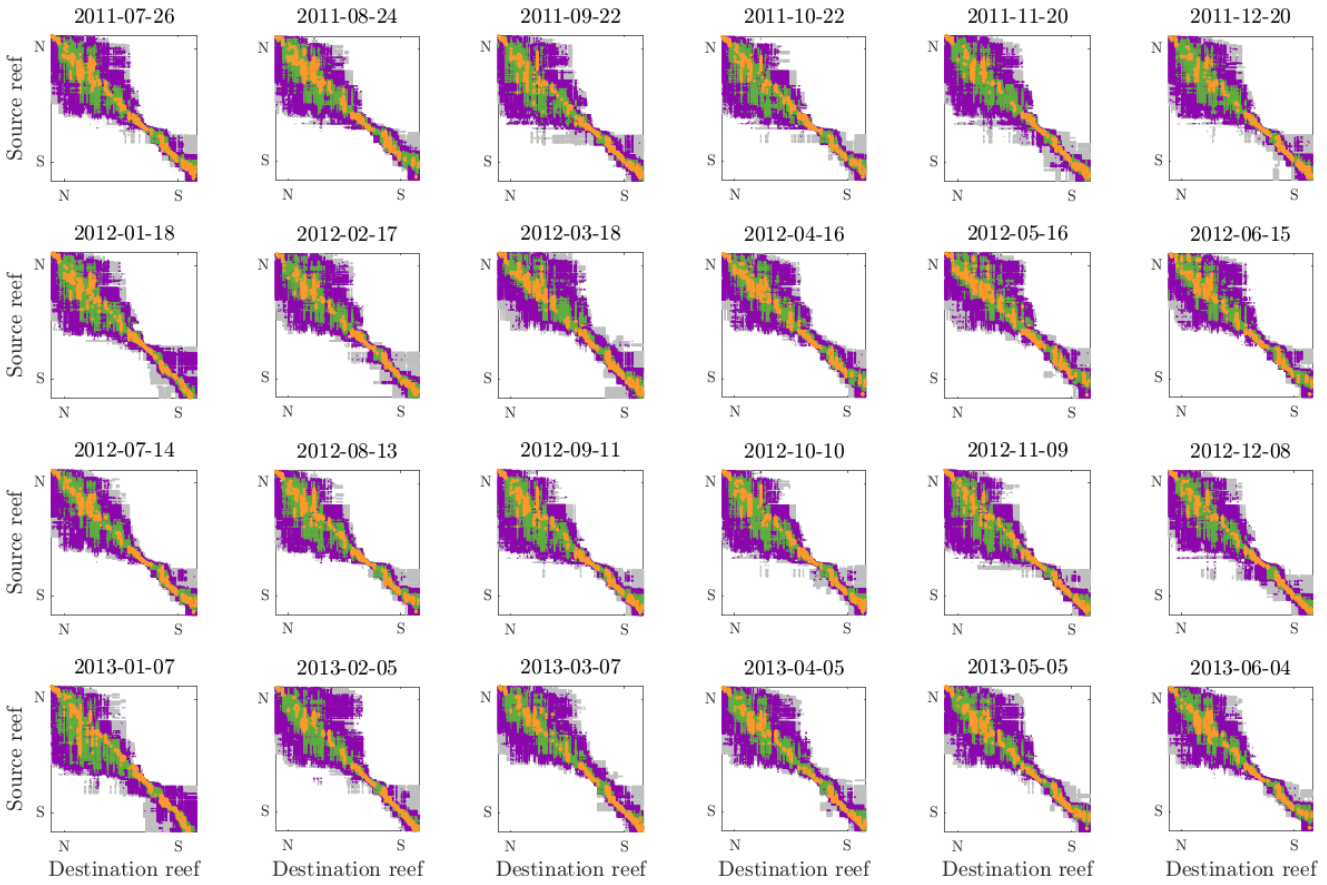

Figure S6: Visualisation of the connectivity matrices for each of the 24 months simulated by the biophysical larval dispersal model. Rows show the source reef, columns show the destination reef. Each connection is coloured as either strong (orange = top 0.2%), moderate (green = top 5%), or weak (purple = top 50%). The remainder of connections are grey, with white areas indicating no dispersal connection.

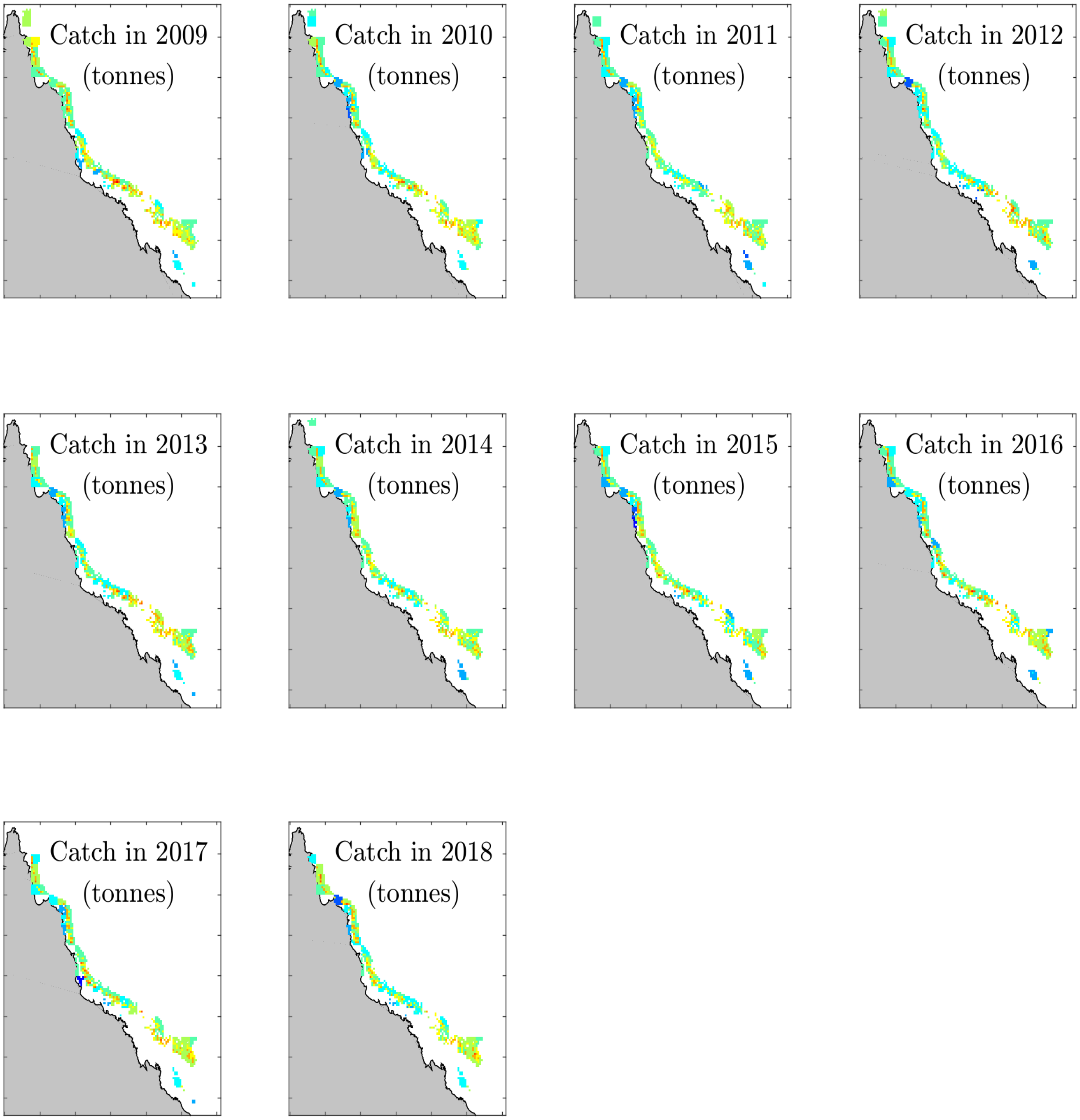

Figure S7: Reported commercial catch in each reporting grid square for the years relevant to the study. Blue regions have the lowest catch, with yellow and red regions reporting the highest catches. Blank regions have no reported catch.

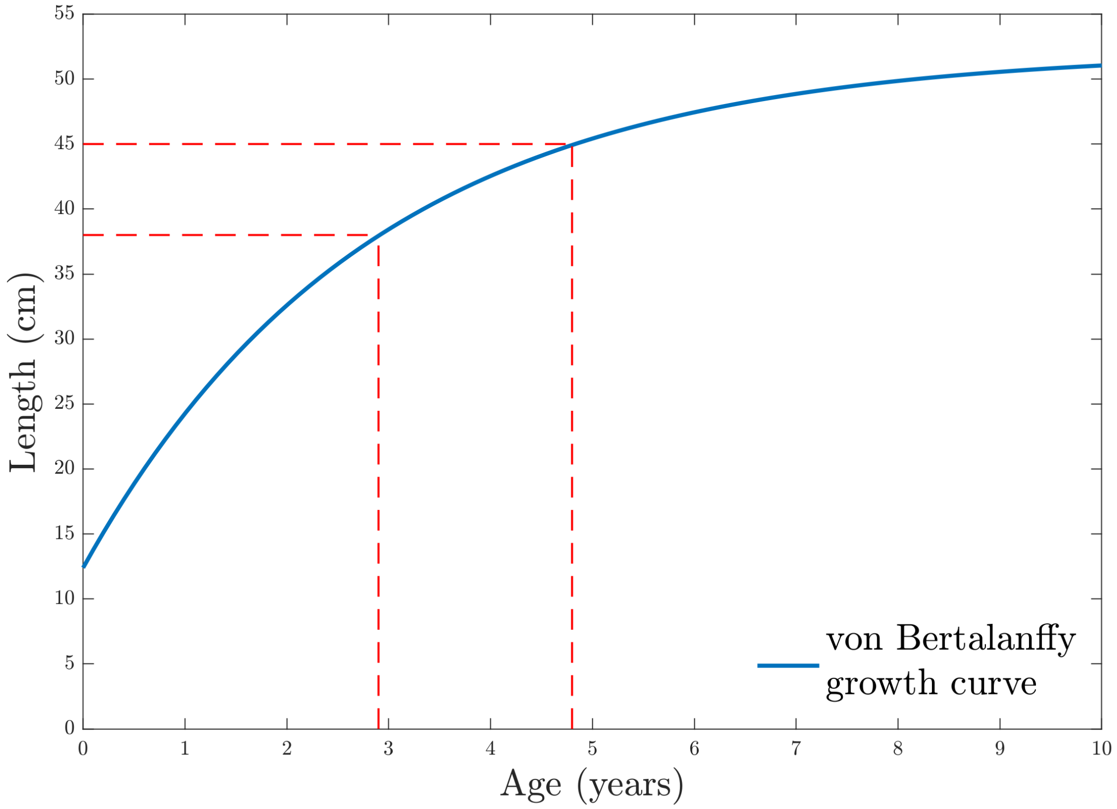

Figure S8: von Bertalanffy growth curve for *P. leopardus*. Horizontal red lines show the range of lengths targeted by the commercial fishery, with the corresponding ages indicated by the vertical red lines.

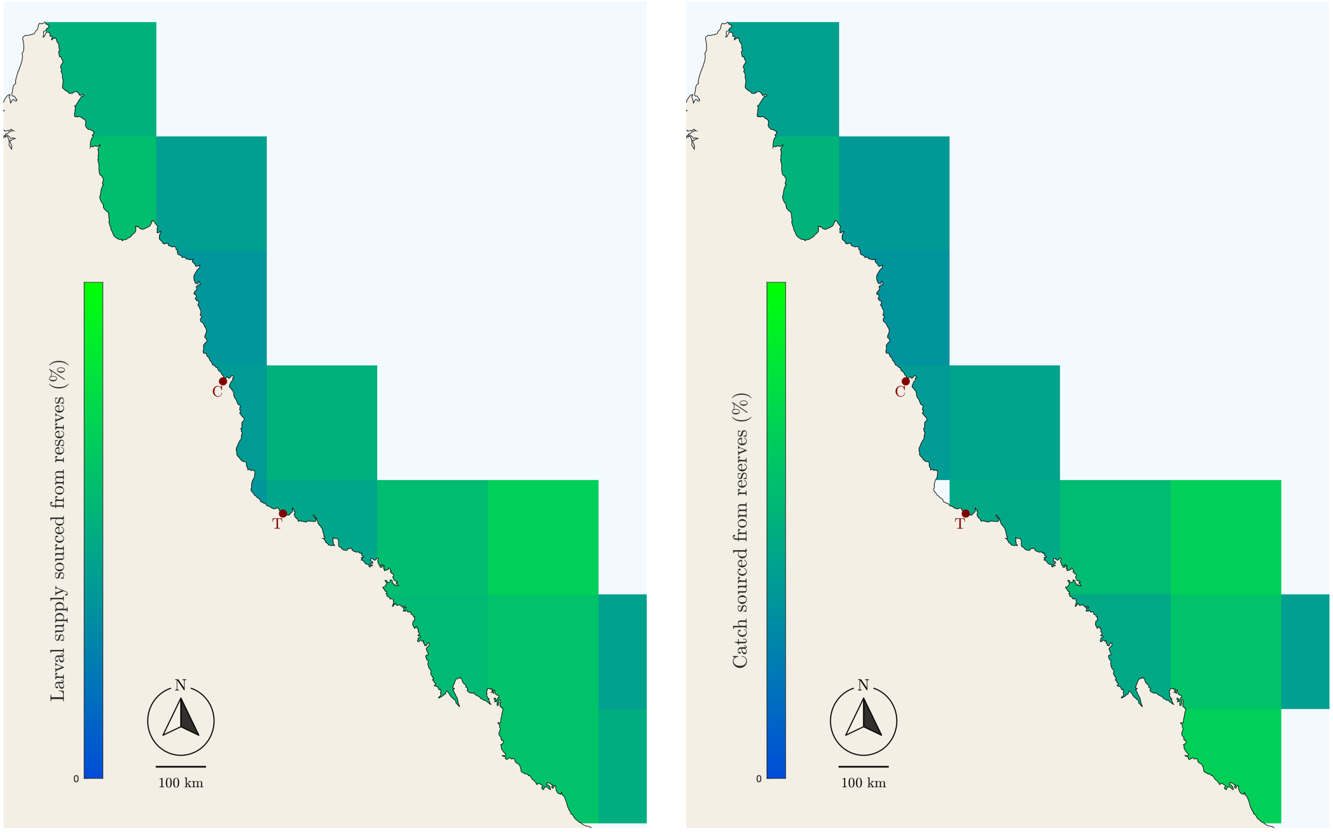

Figure S9: Regionally averaged spatial variation in the contribution of the GBR's marine reserve network to larval settlement (left) and commercial catches (right). Grid squares are colored by the mean proportion of settling larvae that are sourced from marine reserves, within their domain. The cities of Townsville (T) and Cairns (C) are shown.

REFERENCES AND NOTES

1. G. J. Edgar, R. D. Stuart-Smith, T. J. Willis, S. Kininmonth, S. C. Baker, S. Banks, N. S. Barrett, M. A. Becerro, A. T. F. Bernard, J. Berkhout, C. D. Buxton, S. J. Campbell, A. T. Cooper, M. Davey, S. C. Edgar, G. Försterra, D. E. Galván, A. J. Irigoyen, D. J. Kushner, R. Moura, P. E. Parnell, N. T. Shears, G. Soler, E. M. A. Strain, R. J. Thomson, Global conservation outcomes depend on marine protected areas with five key features. *Nature* **506**, 216–220 (2014).

2. S. E. Lester, B. S. Halpern, K. Grorud-Colvert, J. Lubchenco, B. I. Ruttenberg, S. D. Gaines, S. Airamé, R. R. Warner, Biological effects within no-take marine reserves: A global synthesis. *Mar. Ecol. Prog. Ser.* **384**, 33–46 (2009).

3. S. D. Gaines, C. White, M. H. Carr, S. R. Palumbi, Designing marine reserve networks for both conservation and fisheries management. *Proc. Natl. Acad. Sci. U.S.A.* **107**, 18286–18293 (2010).

4. R. Goñi, R. Hilborn, D. Díaz, S. Mallol, S. Adlerstein, Net contribution of spillover from a marine reserve to fishery catches. *Mar. Ecol. Prog. Ser.* **400**, 233–243 (2010).

5. M. A. MacNeil, N. A. J. Graham, J. E. Cinner, S. K. Wilson, I. D. Williams, J. Maina, S. Newman, A. M. Friedlander, S. Jupiter, N. V. C. Polunin, T. R. McClanahan, Recovery potential of the world's coral reef fishes. *Nature* **520**, 341–344 (2015).

6. D. Díaz, S. Mallol, A. M. Parma, R. Goni, A 25-year marine reserve as proxy for the unfished condition of an exploited species. *Biol. Conserv.* **203**, 97–107 (2016).

7. C. P. Lavin, G. P. Jones, D. H. Williamson, H. B. Harrison, Minimum size limits and the reproductive value of numerous, young, mature female fish. *Proc. Roy. Soc. Ser. B* **288,** 20202714 (2021).

8. R. A. Abesamis, G. R. Russ, Density-dependent spillover from a marine reserve: Long-term evidence. *Ecol. Appl.* **15**, 1798–1812 (2005).

9. S. Manel, N. Loiseau, M. Andrello, K. Fietz, R. Goñi, A. Forcada, P. Lenfant, S. Kininmonth, C. Marcos, V. Marques, S. Mallol, A. Pérez-Ruzafa, C. Breusing, O. Puebla, D. Mouillot, Long-distance benefits of marine reserves: Myth or reality? *Trends Ecol. Evol.* **34**, 342–354 (2019).

10. M. Bode, J. N. Sanchirico, P. R. Armsworth, Returns from matching management resolution to ecological variation in a coral reef fishery. *Proc. Roy. Soc. Ser B* **283**, 20152828 (2016).

11. N. C. Krueck, G. N. Ahmadia, H. P. Possingham, C. Riginos, E. A. Treml, P. J. Mumby, Marine reserve targets to sustain and rebuild unregulated fisheries. *PLOS Biol.* **15**, e2000537 (2017).

12. K. A. Catalano, A. G. Dedrick, M. R. Stuart, J. B. Puritz, H. R. Montes Jr., M. L. Pinsky, Quantifying dispersal variability among nearshore marine populations. *Mol. Ecol.* **30**, 2366–2377 (2020).

13. M. J. Emslie, M. Logan, D. H. Williamson, A. M. Ayling, M. A. MacNeil, D. Ceccarelli, A. J. Cheal, R. D. Evans, K. A. Johns, M. J. Jonker, I. R. Miller, K. Osborne, G. R. Russ, H. P. A. Sweatman, Expectations and outcomes of reserve network performance following re-zoning of the great barrier reef marine park. *Curr. Biol.* **25**, 983–992 (2015).

14. A. J. Frisch, D. S. Cameron, M. S. Pratchett, D. H. Williamson, A. J. Williams, A. D. Reynolds, A. S. Hoey, J. R. Rizzari, L. Evans, B. Kerrigan, G. Muldoon, D. J. Welch, J. P. A. Hobbs, Key aspects of the biology, fisheries and management of Coral grouper. *Rev. Fish Biol. Fish.* **26**, 303–325 (2016).

15. R. K. Cowen, in *Coral Reef Fishes: Dynamics and Diversity in a Complex Ecosystem*, P. F. Sale, Ed. (Academic Press, 2002), pp. 149–170.

16. M. Bode, J. M. Leis, L. B. Mason, D. H. Williamson, H. B. Harrison, S. Choukroun, G. P. Jones, Successful validation of a larval dispersal model using genetic parentage data. *PLOS Biol.* **17**, e3000380 (2019).

17. H. B. Harrison, M. Bode, D. H. Williamson, M. L. Berumen, G. P. Jones, A connectivity portfolio effect stabilizes marine reserve performance. *Proc. Natl. Acad. Sci. U.S.A.* **117**, 25595–25600 (2020).

18. R. A. Abesamis, P. Saenz-Agudelo, M. L. Berumen, M. Bode, C. R. L. Jadloc, L. A. Solera, C. L. Villanoy, L. P. C. Bernardo, A. C. Alcala, G. R. Russ, Reef-fish larval dispersal patterns validate no-take marine reserve network connectivity that links human communities. *Coral Reefs* **36**, 791–801 (2017).

19. D. H. Williamson, H. B. Harrison, G. R. Almany, M. L. Berumen, M. Bode, M. C. Bonin, S. Choukroun, P. J. Doherty, A. J. Frisch, P. Saenz-Agudelo, G. P. Jones, Large-scale, multidirectional larval connectivity among coral reef fish populations in the Great Barrier Reef Marine Park. *Mol. Ecol.* **25**, 6039–6054 (2016).

20. A. B. Campbell, A. R. Fox, J. D. Zieth, "Stock assessment of the Queensland east coast common coral trout (*Plectropomus leopardus*) fishery" (Queensland Government, Brisbane, Australia, 2019).

21. G. R. Almany, M. L. Berumen, S. R. Thorrold, S. Planes, G. P. Jones, Local replenishment of coral reef fish populations in a marine reserve. *Science* **316**, 742–744 (2007).

22. G. R. Almany, S. Planes, S. R. Thorrold, M. L. Berumen, M. Bode, P. Saenz-Agudelo, M. C. Bonin, A. J. Frisch, H. B. Harrison, V. Messmer, G. B. Nanninga, M. A. Priest, M. Srinivasan, T. Sinclair-Taylor, D. H. Williamson, G. P. Jones, Larval fish dispersal in a coral-reef seascape. *Nat. Ecol. Evol.* **1**, 148 (2017).

23. L. Fernandes, J. Day, A. Lewis, S. Slegers, B. Kerrigan, D. Breen, D. Cameron, B. Jago, J. Hall, D. Lowe, J. Innes, J. Tanzer, V. Chadwick, L. Thompson, K. Gorman, M. Simmons, B. Barnett, K. Sampson, G. De'ath, B. Mapstone, H. Marsh, H. Possingham, I. Ball, T. Ward, K. Dobbs, J. Aumend, D. Slater, K. Stapleton, Establishing representative no-take areas in the Great Barrier Reef: Large-scale implementation of theory on marine protected areas. *Cons. Biol.* **19**, 1733–1744 (2005).

24. M. Bode, J. C. Day, "The Great Barrier Reef - Systematically protecting connectivity without connectivity data," in *IUCN Guidance for Conserving Connectivity through Ecological Corridors and Networks* (IUCN, Gland, 2020).

25. G. R. Almany, S. R. Connolly, D. D. Heath, J. D. Hogan, G. P. Jones, L. J. McCook, M. Mills, R. L. Pressey, D. H. Williamson, Connectivity, biodiversity conservation and the design of marine reserve networks for coral reefs *Coral Reefs* **28**, 339–351 (2009).

26. L. Thiault, L. Kernaléguen, C. W. Osenberg, T. Lison de Loma, Y. Chancerelle, G. Siu, J. Claudet, Ecological evaluation of a marine protected area network: A progressive-change BACIPS approach. *Ecosphere* **10**, e02576 (2019).

27. W. J. Fletcher, R. E. Kearney, B. S. Wise, W. J. Nash, Large-scale expansion of no-take closures within the Great Barrier Reef has not enhanced fishery production. *Ecol. Appl.* **25**, 1187–1196 (2015).

28. P. J. Mumby, I. A. Elliott, C. M. Eakin, W. Skirving, C. B. Paris, H. J. Edwards, S. Enríquez, R. Iglesias-Prieto, L. M. Cherubin, J. R. Stevens, Reserve design for uncertain responses of coral reefs to climate change. *Ecol. Lett.* **14**, 132–140 (2011).

29. D. M. Kaplan, L. W. Botsford, Effects of variability in spacing of coastal marine reserves on fisheries yield and sustainability. *Can. J. Fish. Aquat. Sci.*. **62**, 905–912 (2005).

30. G. M. Leigh, A. R. Fox, "Stock assessment of the Queensland east coast common coral grouper (*Plectropomus leopardus*) fishery" (Queensland Government, Brisbane, Australia, 2014).

31. H. B. Harrison, M. L. Berumen, P. Saenz-Agudelo, E. Salas, D. H. Williamson, G. P. Jones, Widespread hybridization and bidirectional introgression in sympatric species of coral reef fish. *Mol. Ecol.* **26**, 5692–5704 (2017).

32. B. P. Ferreira, G. R. Russ, Age, growth and mortality of the inshore coarl trout *Plectropomus maculatus* (Pisces: Serranidae) from the Central Great Barrier Reef, Australia. *Aust. J. Mar. Freshwater Res.* **43**, 1301–1312 (1992)

33. P. C. Heemstra, J. E. Randall, *FAO Species Catalogue. Vol. 16. Groupers of the World (Family Serranidae, Subfamily Epinephelinae)* (FAO, 1993).

34. B. D. Mapstone, C. R. Davies, L. R. Little, A. E. Punt, A. D. M. Smith, F. Pantus, D. C. Lou, A. J. Williams, A. Jones, A. M. Ayling, G. R. Russ, A. D. McDonald, "The effects of line fishing on the Great Barrier Reef and evaluations of alternative potential management strategies" (CRC Reef Research Centre, Townsville, 2004).

35. B. P. Ferreira, G. R. Russ, Age validation and estimation of growth rate of the coral trout, *Plectropomus leopardus* (Lacepède 1802) from Lizard Island, Northern Great Barrier Reef. *Fish. Bull.* **92**, 46–57 (1993).

36. C. Mellin, C. J. A. Bradshaw, M. Meekan, M. J. Caley, Environmental and spatial predictors of species richness and abundance in coral reef fishes. *Glob. Ecol. Biogeogr.* **19**, 212–222 (2010).

37. A.B. Carter, A.J. Williams, G.R. Russ, Increased accuracy of batch fecundity estimates using oocyte stage ratios inPlectropomus leopardus. *J. Fish Biol.* **75**, 716–722 (2009).

38. D. R. Barneche, D. R. Robertson, C. R. White, D. J. Marshal, Fish reproductive-energy output increases disproportionately with body size. *Science* **360**, 642–645 (2018).

39. M. K. James, P. R. Armsworth, L. B. Mason, L. Bode, The structure of reef fish metapopulations: Modelling larval disperal and retention patterns. *Proc. R. Soc. Lond. B* **269**, 2079–2086 (2002).

40. J.L. Luick, L. Mason, T. Hardy, M. J. Furnas, Circulation in the Great Barrier Reef Lagoon using numerical tracers and in situ data. *Cont. Shelf Res.* **27,** 757–778 (2007).

41. E. Staaterman, C.B. Paris, Modelling larval fish navigation: The way forward. *ICES J. Mar. Sci.* **71**, 918–924 (2014).

42. L. Goggin, "Line fishing on the Great Barrier Reef: Current state of knowledge" (CRC Reef Research Centre, Townsville, 2002).
